# Supplementary material for: Eight Years of African Swine Fever in the Baltic States: Epidemiological Reflections
Source: Pathogens. 2022 Jun 20;11(6):711. doi: 10.3390/pathogens11060711 (PMC9230151; doi:10.3390/pathogens11060711)
Supplement: Supplementary file 1 [file pathogens-11-00711-s001.zip › pathogens-1752093-supplementary.pdf]

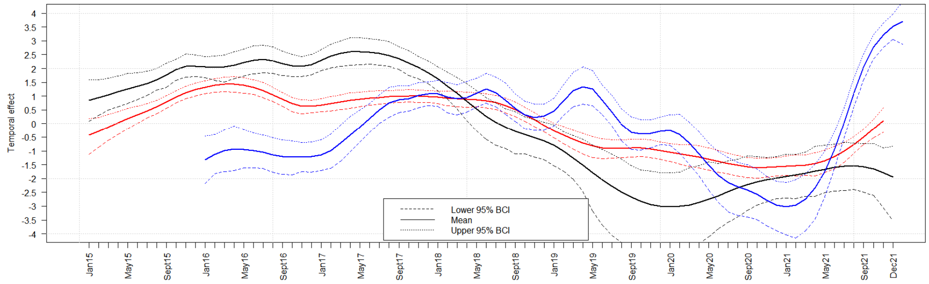

Figure S1: Median temporal effect on the logit prevalence for hunted wild boar that tested positive for ASFV in Estonia (black lines), Latvia (red lines) and Lithuania (blue lines). The 95% Bayesian credible intervals (BCIs, dashed lines) are indicated for each country in the respective color.

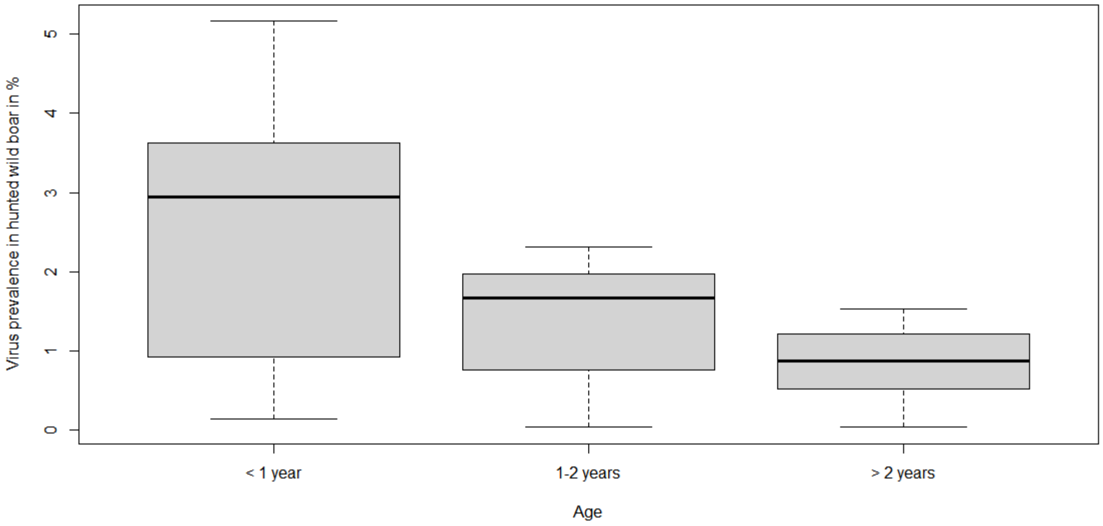

Figure S2: ASFV prevalence estimates in hunted wild boar from Estonia, Latvia and Lithuania in the three different age classes. The horizontal lines that form the top of the boxes illustrate the 75th percentile. The horizontal lines that form the bottom represent the 25th percentile. The horizontal lines that intersect the box are the estimated median ASFV prevalence in hunted wild boar. Whiskers represent maximum and minimum values that are no more than 1.5 times the span of the interquartile range.

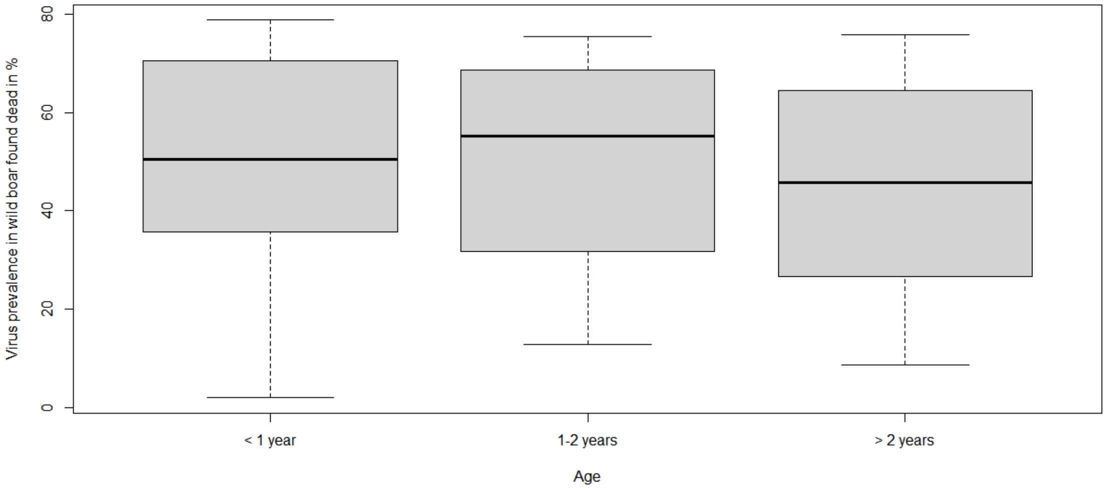

Figure S3: ASFV prevalence estimates in wild boar found dead from Estonia, Latvia and Lithuania in the three different age classes. The horizontal lines that form the top of the boxes illustrate the 75th percentile. The horizontal lines that form the bottom represent the 25th percentile. The horizontal lines that intersect the box are the estimated median ASFV prevalence in wild boar found dead. Whiskers represent maximum and minimum values that are no more than 1.5 times the span of the interquartile range.

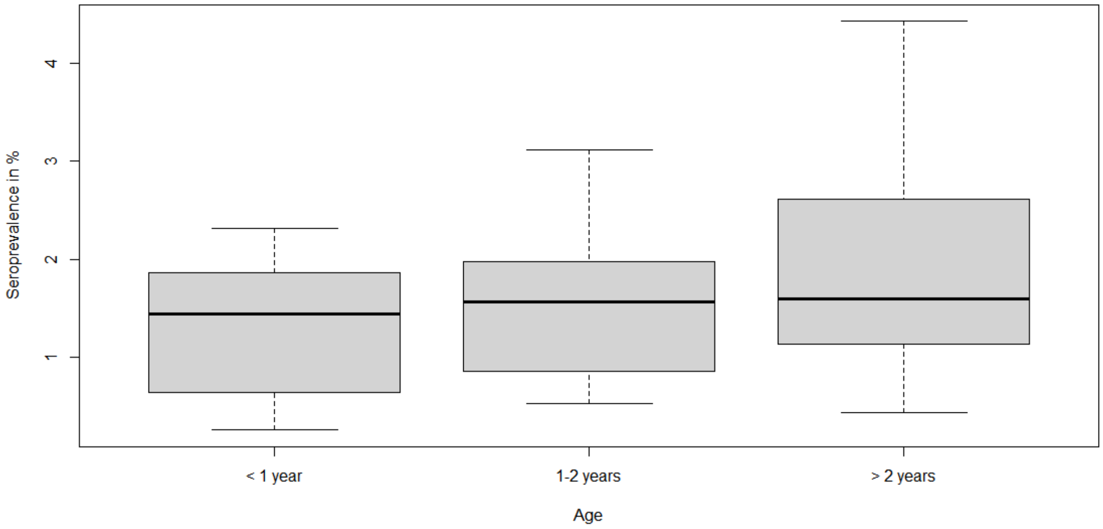

Figure S4: Seroprevalence estimates in hunted wild boar from Estonia, Latvia and Lithuania in the three different age classes. The horizontal lines that form the top of the boxes illustrate the 75th percentile. The horizontal lines that form the bottom are the 25th percentile. The horizontal lines that intersect the box are the estimated median seroprevalence in hunted wild boar. Whiskers represent maximum and minimum values that are no more than 1.5 times the span of the interquartile range and the open circles represent outliers, which are single values greater or less than the extremes indicated by the whiskers.

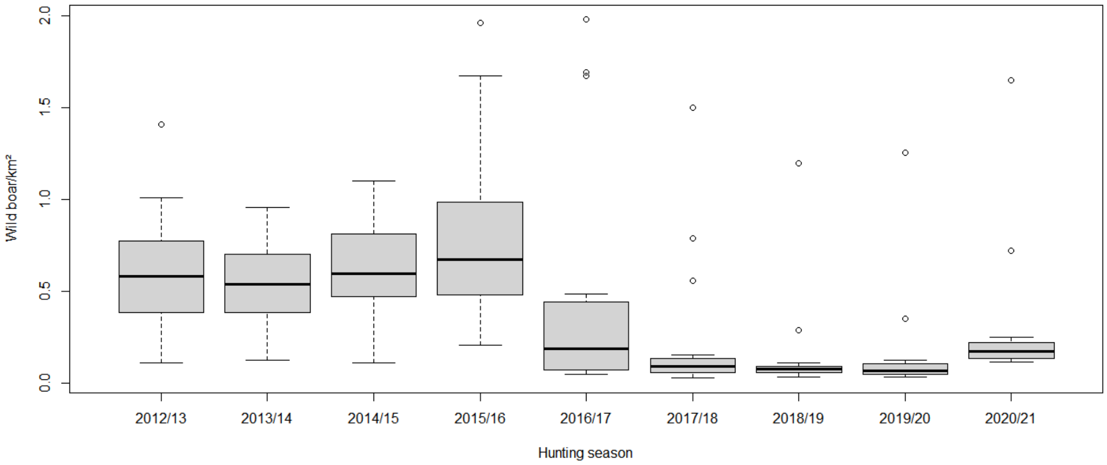

Figure S5: Estimated Estonian wild boar population density (wild boar/km²) per hunting season. The horizontal lines that form the top of the boxes illustrate the 75th percentile. The horizontal lines that form the bottom indicate the 25th percentile. The horizontal lines that intersect the box represent the median number of wild boar per square kilometer. Whiskers indicate maximum and minimum values that are no more than 1.5 times the span of the interquartile range. Open circles represent outliers, which are single values greater or less than the extremes indicated by the whiskers.

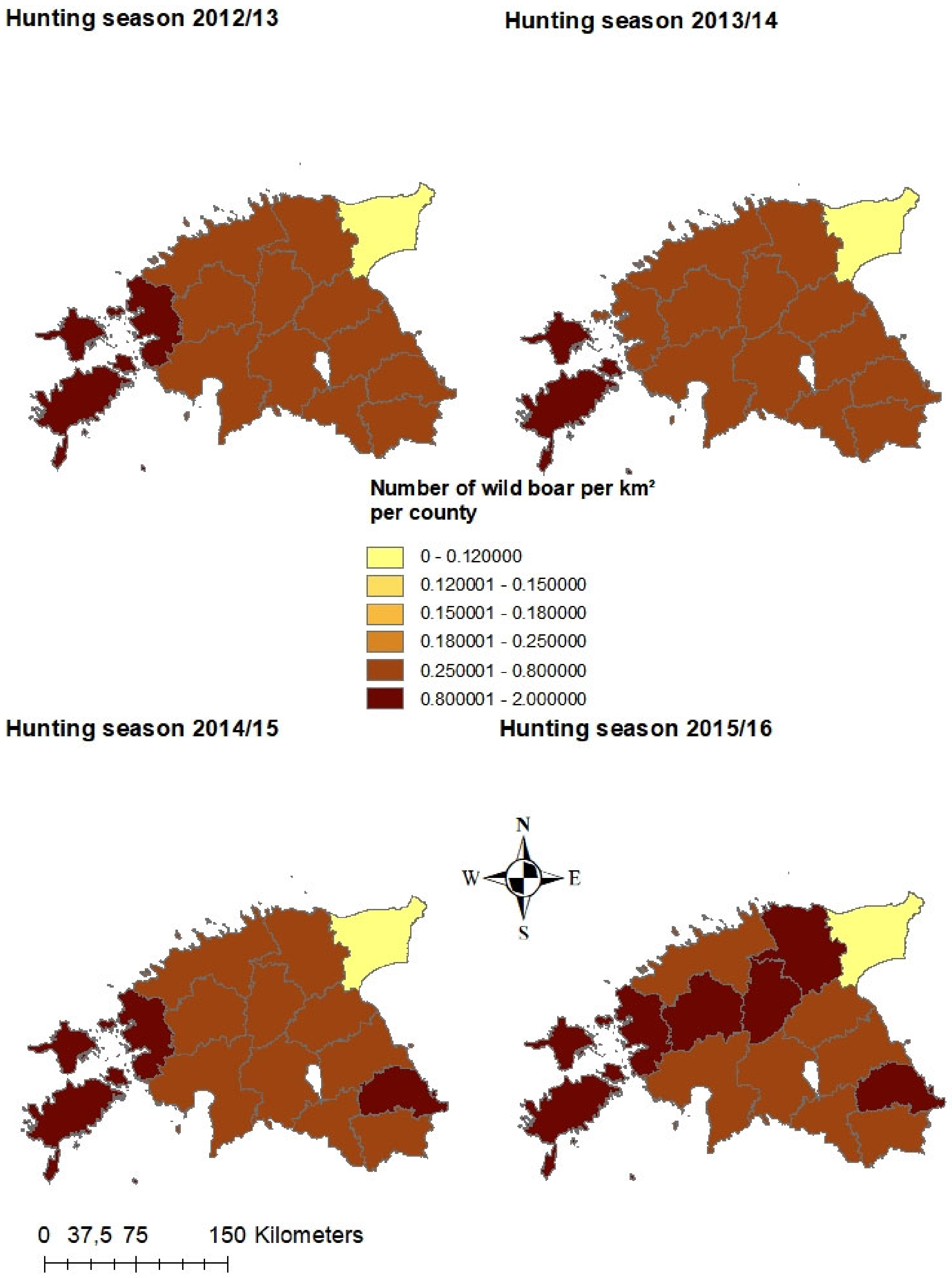

Figure S6: Estimated numbers of wild boar/km² in Estonian counties in the hunting seasons 2012/13-2015/16.

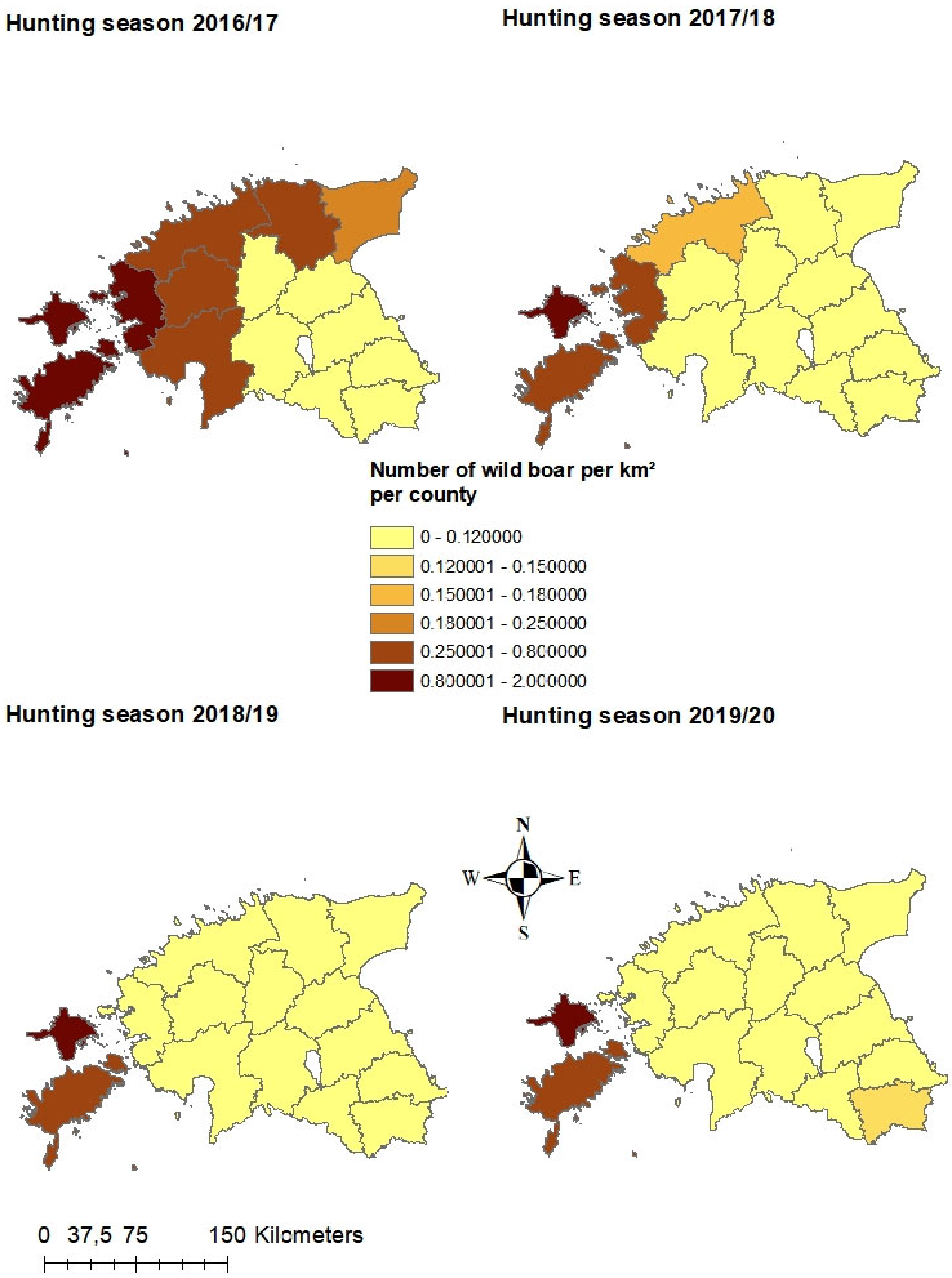

Figure S7: Estimated numbers of wild boar/km² in Estonian counties in the hunting seasons 2016/17-2019/20.

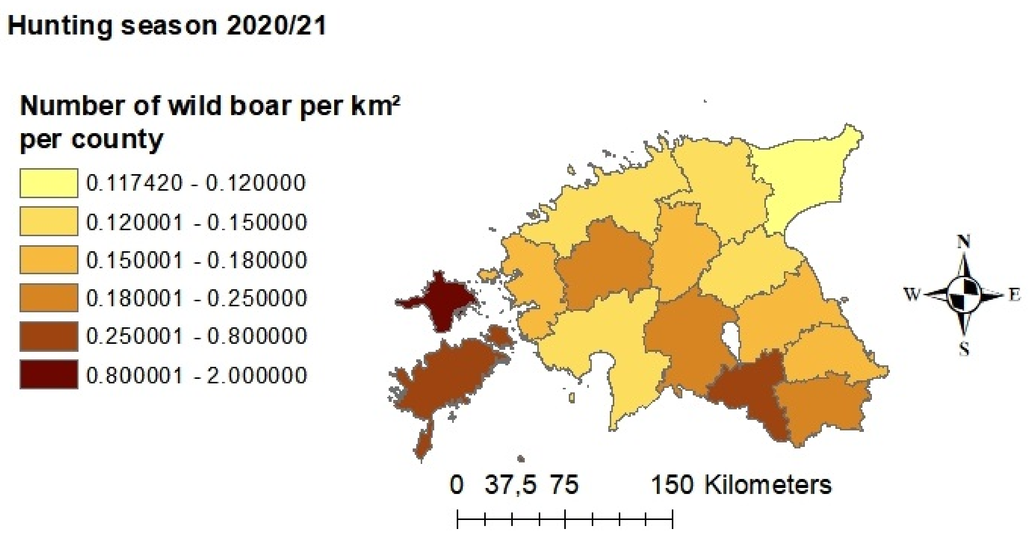

Figure S8: Estimated numbers of wild boar/km² in Estonian counties in the hunting season 2020/21.

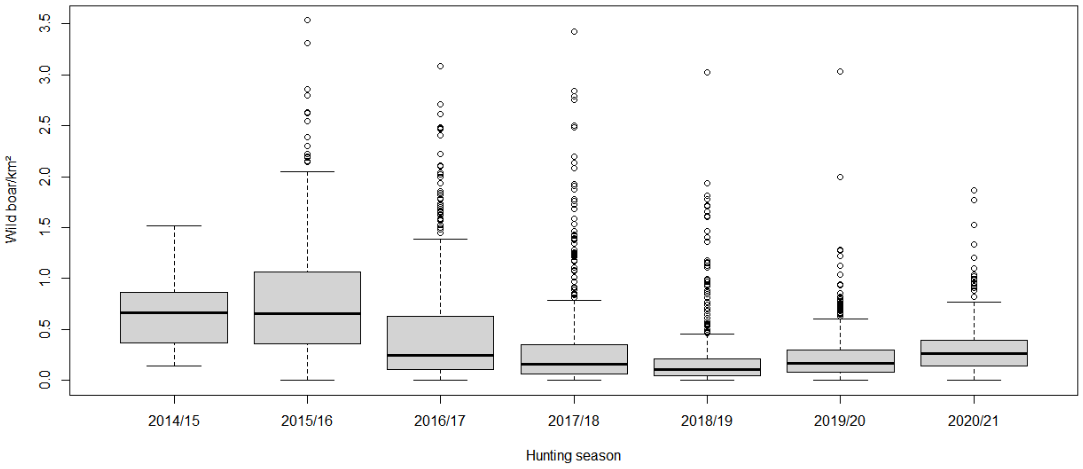

Figure S9: Estimated Latvian wild boar population density (wild boar/km²) per hunting season. The horizontal lines that form the top of the boxes illustrate the 75th percentile. The horizontal lines that form the bottom indicate the 25th percentile. The horizontal lines that intersect the box represent the median number of wild boar per square kilometer. Whiskers indicate maximum and minimum values that are no more than 1.5 times the span of the interquartile range. Open circles represent outliers, which are single values greater or less than the extremes indicated by the whiskers.

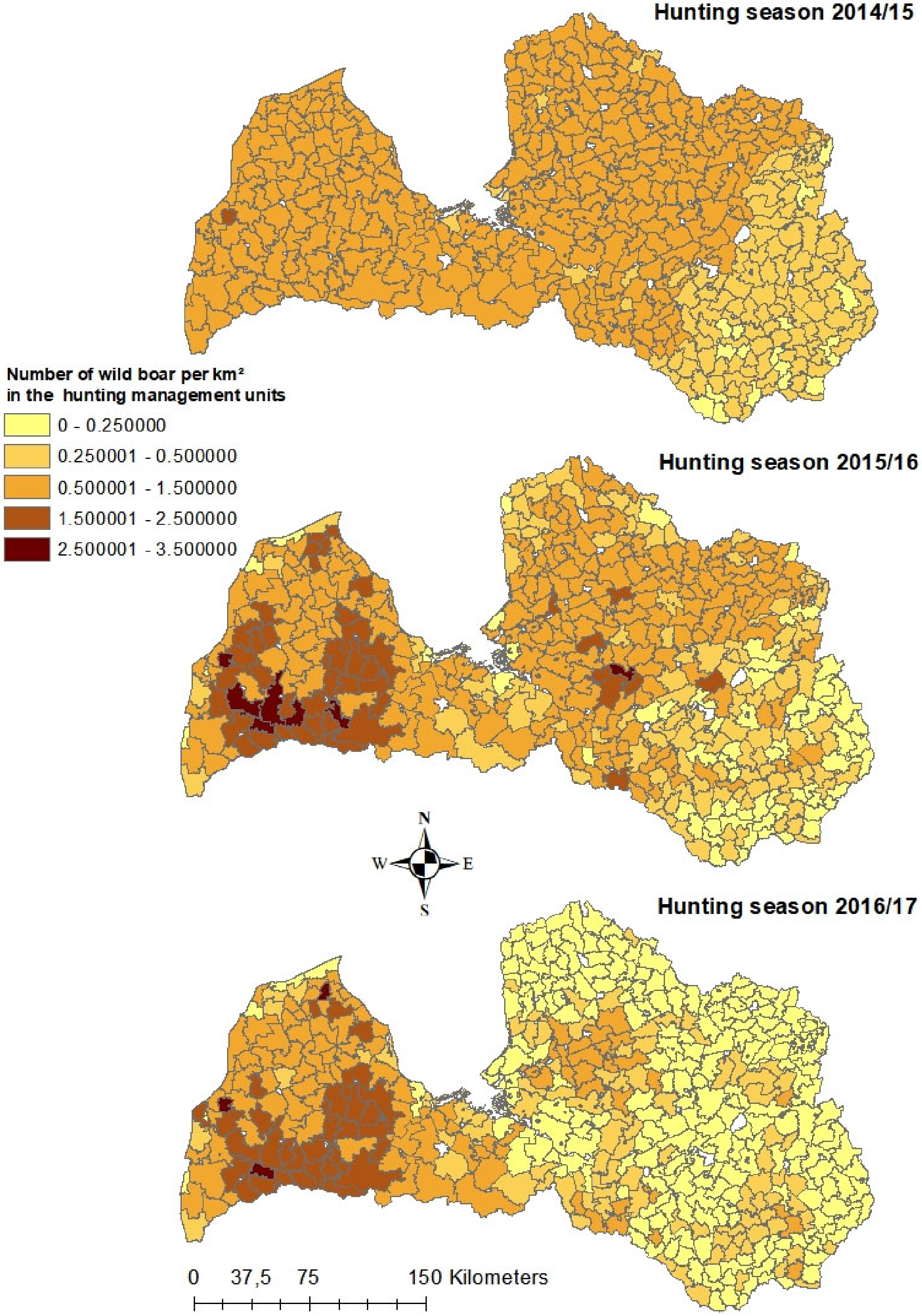

Figure S10: Estimated numbers of wild boar/km² in Latvian hunting management units in the hunting seasons 2014/15-2016/17.

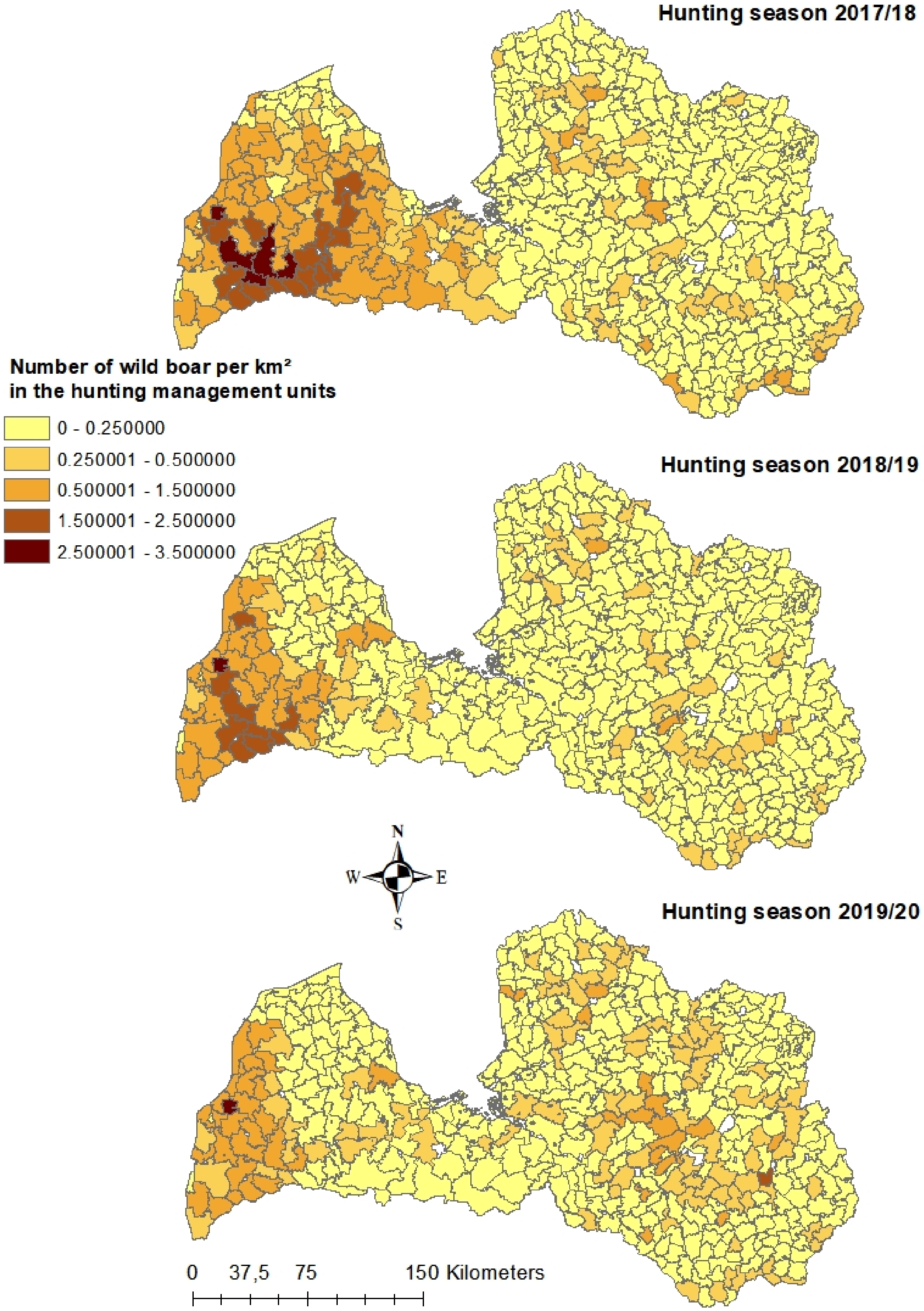

Figure S11: Estimated numbers of wild boar/km² in Latvian hunting management units in the hunting seasons 2017/18-2019/20.

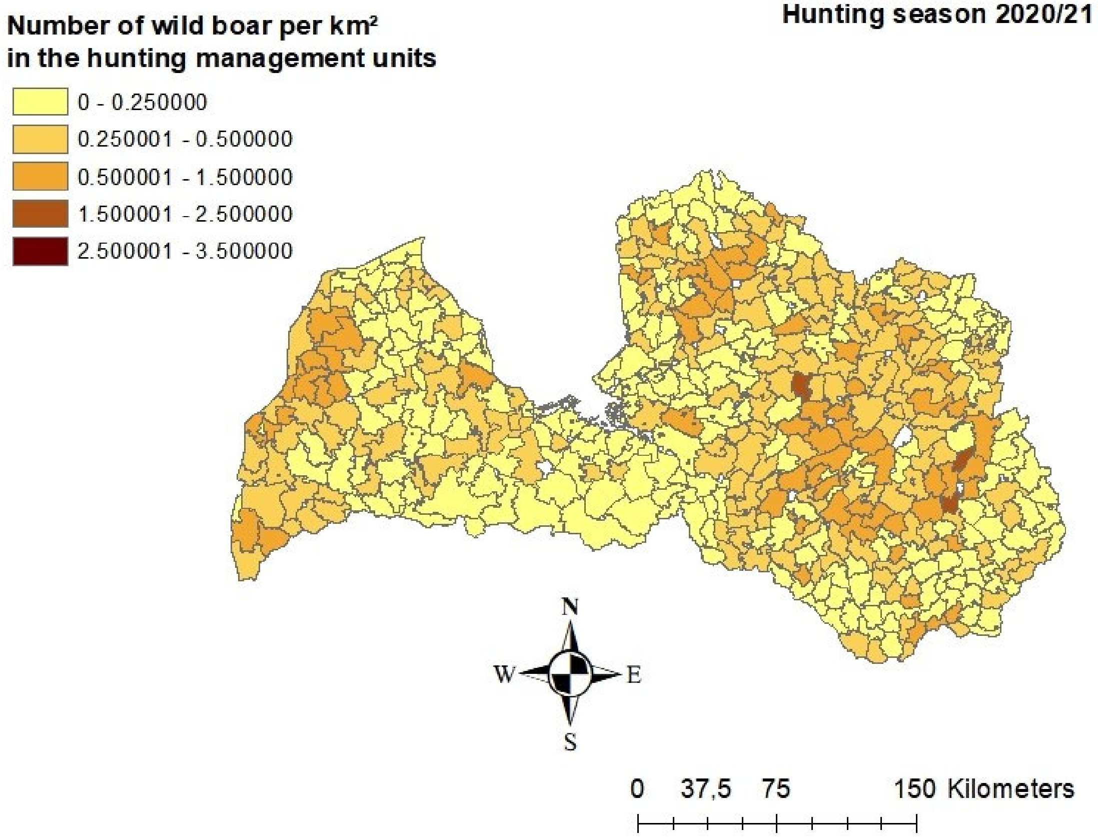

Figure S12: Estimated numbers of wild boar/km² in Latvian hunting management units in the hunting season 2020/21.

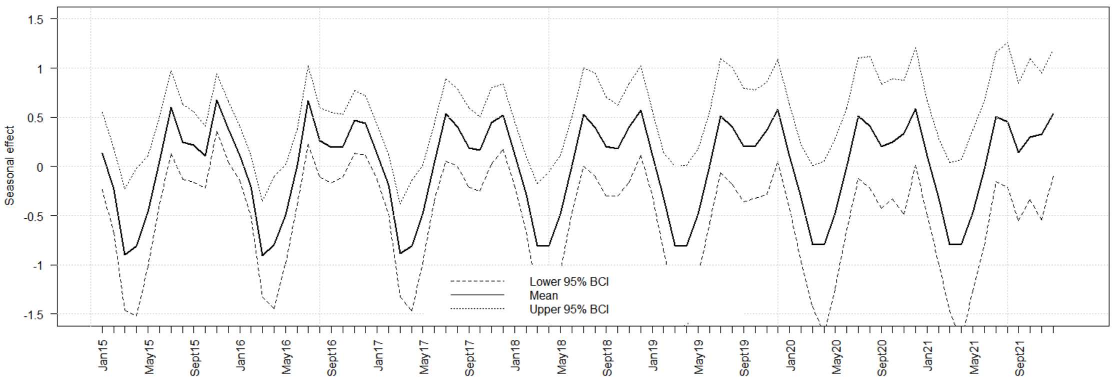

Figure S13: Median seasonal effect of samples obtained from hunted wild boar in Estonia that tested PCR-positive for ASFV, irrespective of the serological result, on the logit prevalence. 95% Bayesian credible intervals (BCI) are indicated.

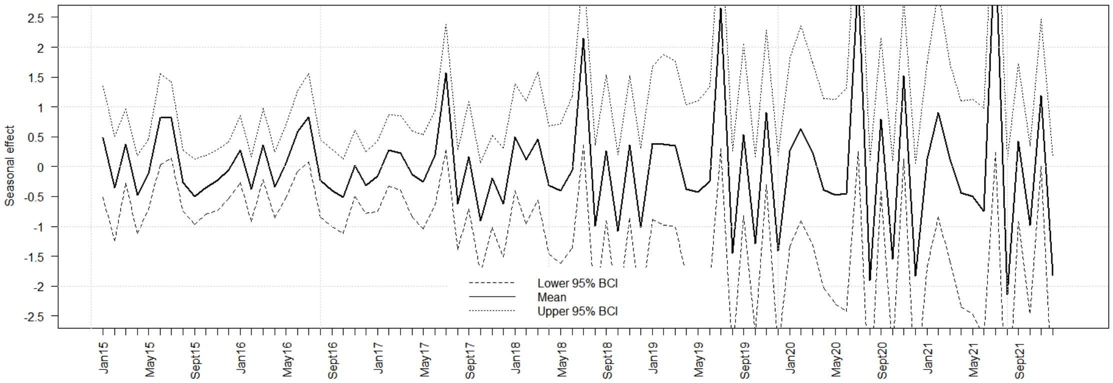

Figure S14: Median seasonal effect of samples obtained from wild boar found dead in Estonia that tested PCR-positive for ASFV, irrespective of the serological result, on the logit prevalence. 95% Bayesian credible intervals (BCI) are indicated.

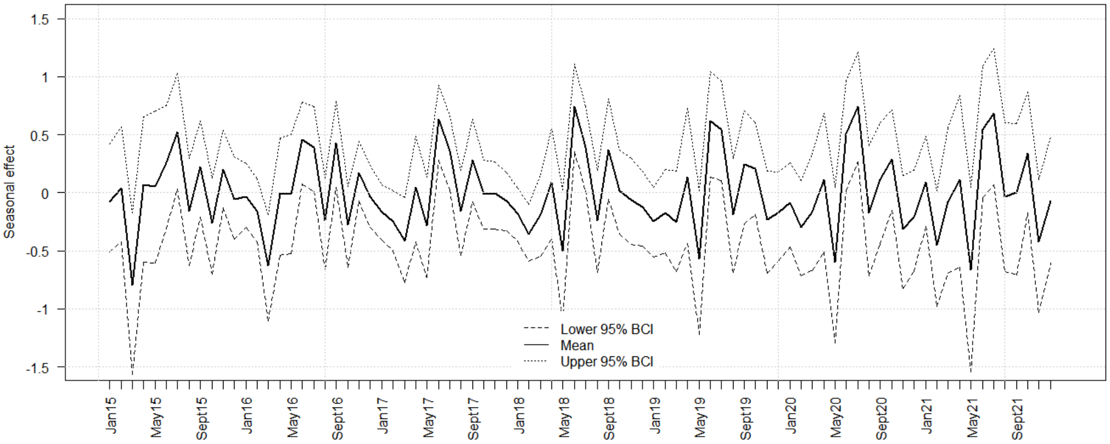

Figure S15: Median seasonal effect of samples obtained from hunted wild boar in Estonia that tested exclusively serologically positive for antibodies to ASFV on the logit prevalence, on the logit prevalence. 95% Bayesian credible intervals (BCI) are indicated.

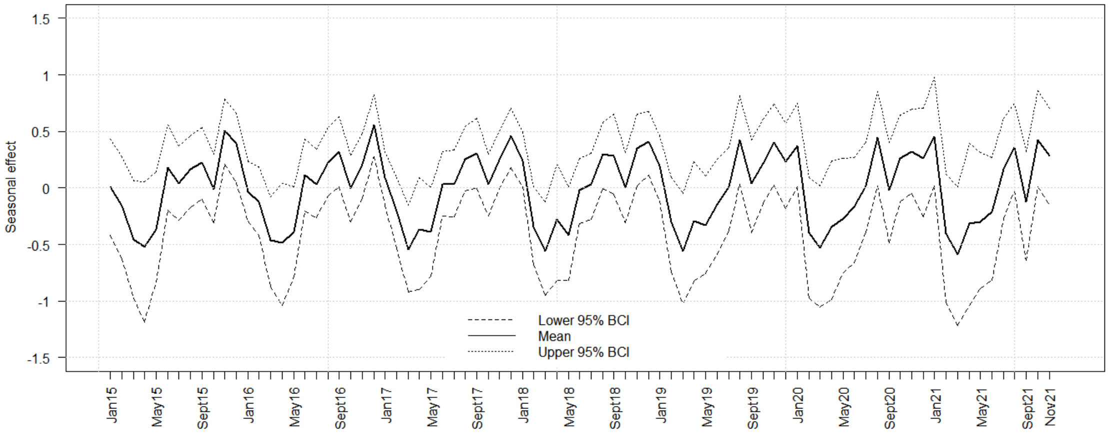

Figure S16: Median seasonal effect of samples obtained from hunted wild boar in Latvia that tested PCR-positive for ASFV, irrespective of the serological result, on the logit prevalence. 95% Bayesian credible intervals (BCI) are indicated.

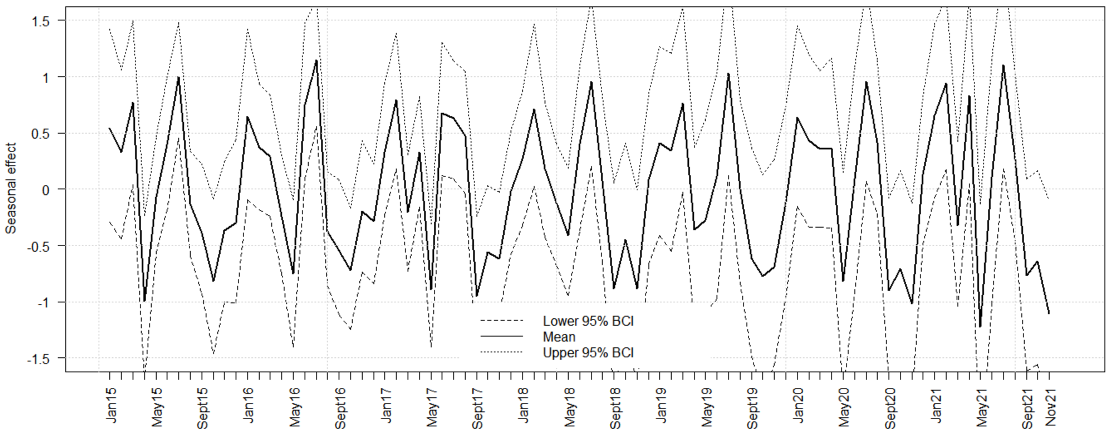

Figure S17: Median seasonal effect of samples obtained from wild boar found dead in Latvia that tested PCR-positive, irrespective of the serological result, on the logit prevalence. 95% Bayesian credible intervals (BCI) are indicated.

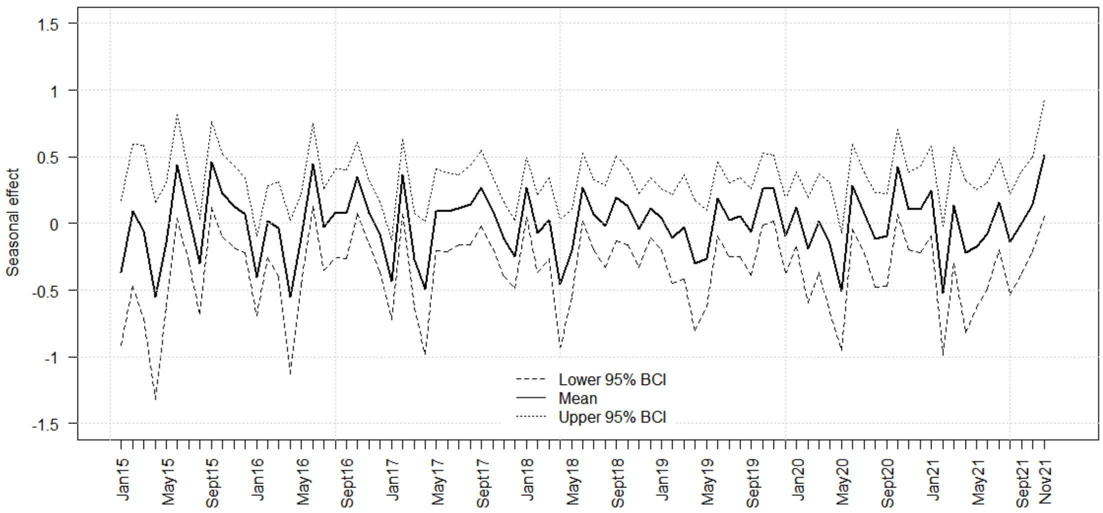

Figure S18: Median seasonal effect of samples obtained from hunted wild boar in Latvia that tested exclusively serologically positive for antibodies to ASFV on the logit prevalence, on the logit prevalence. 95% Bayesian credible intervals (BCI) are indicated.

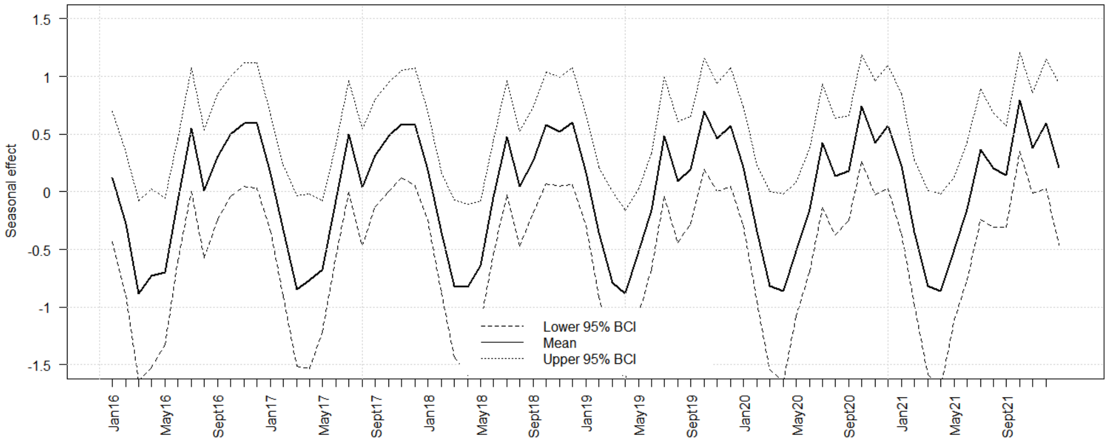

Figure S19: Median seasonal effect of samples obtained from hunted wild boar in Lithuania that tested PCR-positive for ASFV, irrespective of the serological result, on the logit prevalence. 95% Bayesian credible intervals (BCI) are indicated.

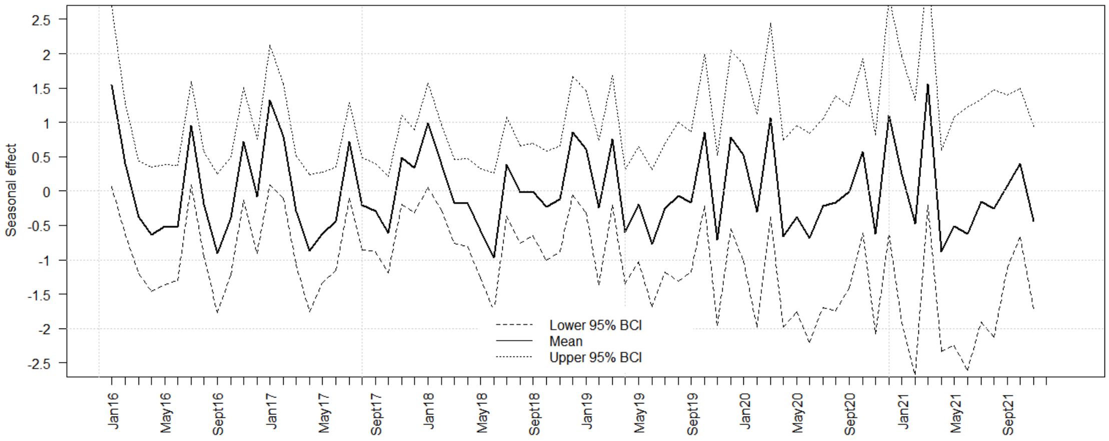

Figure S20: Median seasonal effect of samples obtained from wild boar found dead in Lithuania that tested PCR-positive for ASFV, irrespective of the serological result, on the logit prevalence. 95% Bayesian credible intervals (BCI) are indicated.

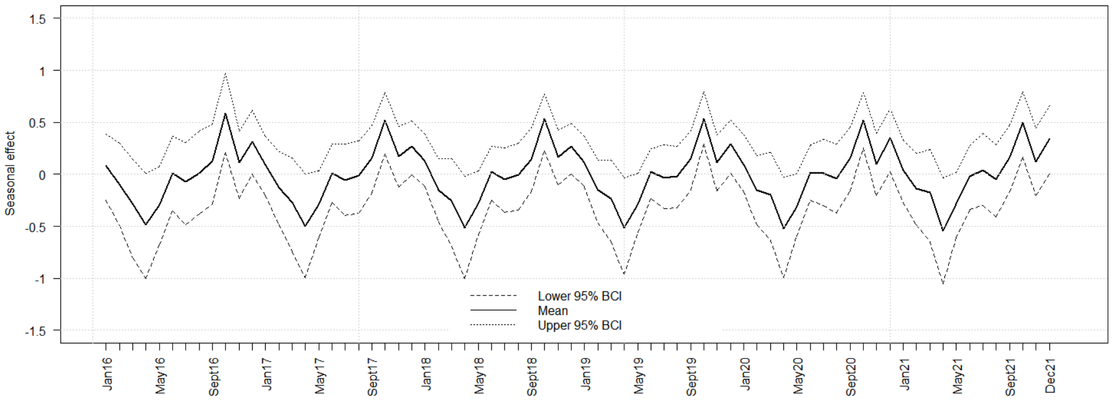

Figure S21: Median seasonal effect of samples obtained from hunted wild boar in Lithuania that tested exclusively serologically positive for antibodies to ASFV on the logit prevalence, on the logit prevalence. 95% Bayesian credible intervals (BCI) are indicated.
